# Survival of cancer patients with pre-existing heart disease

**DOI:** 10.1186/s12885-022-09944-z

**Published:** 2022-08-03

**Authors:** Ciaran O’Neill, David W. Donnelly, Mark Harbinson, Therese Kearney, Colin R. Fox, Gerard Walls, Anna Gavin

**Affiliations:** 1 Northern Ireland Cancer Registry, Belfast, UK; 2 Centre for Public Health, Queens University Belfast, Belfast, UK; 3 School of Medicine, Dentistry and Biomedical Sciences, Queens University Belfast, Belfast, UK; 4 Patrick G. Johnston Centre for Cancer Research, Queen’s University Belfast, Belfast, UK; 5 Cancer Centre Belfast City Hospital, Belfast Health & Social Care Trust, Belfast, UK

**Keywords:** survival, cancer, pre-existing cardiovascular disease

## Abstract

**Background:**

While cancer outcomes have improved over time, in Northern Ireland they continue to lag behind those of many other developed economies. The role of comorbid conditions has been suggested as a potential contributory factor in this but issues of data comparability across jurisdictions has inhibited efforts to explore relationships. We use data from a single jurisdiction of the UK using data from - the Northern Ireland Cancer Registry (NICR), to examine the association between mortality (all-cause and cancer specific) and pre-existing cardiovascular diseases among patients with cancer.

**Materials and Methods:**

All patients diagnosed with cancer (excluding non-melanoma skin cancer) between 2011 and 2014 were identified from Registry records. Those with a pre-existing diagnosis of cardiovascular diseases were identified by record linkage with patient hospital discharge data using ICD10 codes. Survival following diagnosis was examined using descriptive statistics and Cox proportional hazards regression analyses. Analyses examined all-cause mortality and cancer specific mortality for lung, colorectal, breast and prostate cancer. As well as cardiovascular diseases, regression models controlled for age, gender (where appropriate), deprivation (as quintiles), stage at diagnosis and other comorbidities.

**Results:**

Almost 35,000 incident cancer cases were diagnosed during the study period of which approximately 23% had a prior heart condition. The pan-cancer hazard ratio for death in the presence of pre-existing cardiovascular diseases was 1.28 (95% CI: 1.18-1.40). All-cause and cancer specific mortality was higher for patients with cardiovascular diseases across lung, female breast, prostate and colorectal cancer groups after controlling for age, gender (where appropriate), deprivation (as quintiles), stage at diagnosis and other comorbidities.

**Conclusion:**

Pre-existing morbidity may restrict the treatment of cancer for many patients. In this cohort, cancer patients with pre-existing cardiovascular diseases had poorer outcomes than those without cardiovascular diseases. A high prevalence of cardiovascular diseases may contribute to poorer cancer outcomes at a national level.

**Supplementary Information:**

The online version contains supplementary material available at 10.1186/s12885-022-09944-z.

## Background

Evidence exists of variations in five-year survival between countries for all as well as a number of specific cancers [1–7]. A range of factors to explain these differences include stage [8] and/or delays at diagnosis [9], differences in access to optimal treatments [10] and differences in the quality of the data upon which analyses are based [10–12]. While cancer survival has improved in Northern Ireland (NI) over the past 20 or so years [13], it continues to lag behind that of many other high income countries [3], as does that in the UK and Ireland generally [3]. Efforts to improve outcomes are predicated on a clear understanding of the factors that underpin suboptimal performance.

Recent studies have investigated the contribution of differences in patterns of comorbidity to cancer outcomes, and by inference part of the difference in performance between jurisdictions may relate to differences in the burden and pattern of comorbidity [14–16]. Efforts to examine this issue using international data are, however, challenging given differences in coding practices for comorbid conditions across jurisdictions [14]. While studies within single jurisdictions generally show a survival penalty associated with a higher comorbidity burden [15], these often examine only one specific cancer [17–20], limit the age group studied [20] or fail to control for crucial clinically relevant covariates such as socio-economic status [21]. The latter may be of particular importance given the potential for such factors to explain differences in the speed with which medical help is sought/provided [22, 23], eligibility for specific treatments and implications for inequalities in the health care system [24, 25].

The aim of this study was to examine the impact of comorbidity and in particular pre-existing cardiovascular diseases on cancer survival within one jurisdiction in which there existed uniform coding. One previous study has examined this issue albeit in a different population – South Korean – among whom life styles and cancer biology differ from Western populations [26]. While the study by Youn et al [26] produced compelling results important determinants of cancer outcomes such as cancer staging were not available. Given this and the differences between these populations further research on these relationships is warranted. Cardiovascular disease was selected as the focus of this dedicated study because of its high prevalence in NI, and emerging evidence of a link between cardiovascular disease and cancer in terms of their origins [27] and prognosis [28].

## Methods

Data on all patients diagnosed with cancer (excluding non-melanoma skin cancer (NMSC)) in the period 2011 to 2014 were extracted from the NI Cancer Registry (NICR) with relevant cases identified using the International Classification of Diseases (ICD10) codes C00-C97 (excluding C44). Subgroups of patients with the most common cancer types were also identified using ICD10 codes, with C18-C20 used for colorectal cancer, C33-C34 for lung cancer, C50 for breast cancer and C61 for prostate cancer.

Using a unique patient identifier (Health and Care Number), cancer data were linked to a hospital inpatient dataset which included all hospital inpatient admissions in Northern Ireland from 2006 to 2014. Identifying cancer patients with pre-existing cardiovascular diseases was based upon any record of a heart condition in the hospital episode data up to five years prior to their cancer diagnosis, even if that condition was not the main reason for hospital admission. The conditions included in the definition of cardiovascular disease were: congestive heart failure, peripheral vascular disease, cardiac arrhythmias (including atrial fibrillation), valvular disease, pulmonary circulation disorder, other ischaemic heart diseases (including myocardial infarction), venous or arterial embolism and thrombosis, myocarditis, pericarditis and cardiac arrest. Each condition was identified using ICD10 codes selected by clinical opinion and are listed in the Supplementary material.

Using a similar methodology further comorbidities were identified for each cancer patient from the hospital inpatient data. These comorbidities were based upon selected conditions from the Charleson [29] and Elixhauser [30] indices and included cerebrovascular disease, chronic pulmonary disease, hypertension, diabetes, liver disease, renal disease, peptic ulcer, anaemia, neurodegenerative disorders and rheumatoid disorders. The ICD10 codes used to identify these comorbidities were based on those used by Luchtenborg et al [14]. Conditions included in these comorbidity indices of a less serious or a psychiatric nature were omitted in this study as hospital episode data may not fully capture their prevalence.

In addition to the above comorbidities, the presence of a previous cancer was identified from the extracted NICR records. Information on patient gender, age and stage of diagnosis was also available from this source as was area of residence, which was used to assign an area-based deprivation measure based upon the NI Multiple Deprivation Measure [31].

The Kaplan-Meier method was used to conduct survival analysis, with survival time taken to be the time between cancer diagnosis and the earliest between date of death, date when the patient left Northern Ireland, or 31/12/2019 which was the last date of follow up for all patients. Overall, cancer-specific and heart-disease specific survival were estimated based upon death certificate information. For cancer-specific and heart-disease specific analysis, all other causes of death were censored at the date of death. Death certificate only cases (i.e. on whom other data were not available) were excluded from the analysis, and analysis was based upon patients rather than cases - for any patient with more than one cancer diagnosis during 2011-2014 details of the most recent cancer were used.

Using the results from the Kaplan-Meier analysis survival curves were plotted for observed (all-cause) survival and for cumulative deaths from cancer and cardiovascular diseases, each for patients with and without a prior history of cardiovascular diseases. Survival curves were compared using the log-rank test. One and five-year all cause and cancer-specific survival were also estimated along with 95% confidence intervals for those with and without a prior history of cardiovascular diseases. Results are presented for all cancers (ex NMSC) along with lung, colorectal, female breast and prostate cancers sub-groups where sufficient numbers were available.

Given that any reported difference in survival between patients with and without pre-existing cardiovascular diseases may be a result of differences in case mix, a Cox proportional hazard model controlling for a range of covariates was fitted for all cause and cancer specific mortality. In addition to the presence of cardiovascular diseases, included in the model were age, gender (where appropriate), deprivation (as quintiles), stage at diagnosis and other comorbidities, the latter entered as series of dummy variables as either present or not. Results are presented as hazard ratios with 95% confidence intervals.

NICR has ethical approval from the Office of Research Ethics Northern Ireland for collection of the data used in this study. Analysis was conducted in R (version 4.0.5) [32], with the survminer package [33] used to produce cancer survival plots.

## Results

Excluding NMSC there were 34,828 individuals (17,449 male, 17,379 female) diagnosed with cancer in the 2011-2014 period in NI (Table 1). Of these 4,656, 4,748, 4,992 and 4,232 were diagnosed with colorectal, lung, female breast and prostate cancer respectively.

**Table 1:** Patients with cancer and proportion with pre-existing cardiovascular diseaseby cancer type and patient characteristics: All cancer

Characteristic	Number of patients (% with heart disease prior to diagnosis)									
	All cancer (ex NMSC)		Colorectal cancer		Lung cancer		Female breast cancer		Prostate cancer	
**Gender**										
All patients	34,828	22.6%	4,656	25.3%	4,748	32.3%	4,992	10.4%	4,232	22.6%
Male	17,449	27.1%	2,572	27.1%	2,635	35.6%	-	-	4,232	22.6%
Female	17,379	18.2%	2,084	23.0%	2,113	28.2%	4,992	10.4%	-	-
**Age group**										
0-54	6,500	3.6%	560	3.9%	341	8.8%	1,628	1.9%	224	5.8%
55-69	10,812	14.3%	1,417	13.2%	1,510	22.7%	1,647	6.0%	1,733	13.6%
70-79	10,541	29.1%	1,575	29.8%	1,893	36.2%	1,016	16.1%	1,543	25.9%
80+	6,975	43.6%	1,104	45.1%	1,004	47.1%	701	32.0%	732	42.3%
**Deprivation**										
Least deprived (Quintile 1)	6,792	20.3%	918	24.1%	646	33.4%	1,088	9.0%	934	19.7%
Quintile 2	7,038	23.3%	987	27.4%	815	32.8%	1,086	9.4%	929	23.8%
Quintile 3	7,060	23.9%	961	26.8%	931	37.5%	993	11.2%	886	23.4%
Quintile 4	7,215	24.1%	927	26.1%	1,005	34.0%	1,007	11.6%	864	24.3%
Most deprived (Quintile 5)	6,719	21.3%	862	21.6%	1,351	26.5%	818	10.9%	619	22.0%
Unknown	4	-	1	-	0	-	0	-	0	-
**Stage**										
Stage I/II	14,516	17.3%	1,964	25.3%	997	34.3%	3,633	7.9%	2,344	19.2%
Stage III	5,382	20.0%	1,200	21.4%	1,030	29.8%	668	8.8%	729	18.5%
Stage IV	6,816	24.2%	936	20.6%	2,072	28.8%	290	11.4%	699	27.2%
Unknown	8,114	32.5%	556	41.4%	649	44.1%	401	34.7%	460	40.0%
**Comorbidity**										
Previous cancer	1,686	28.1%	287	31.0%	300	33.7%	218	16.1%	133	30.8%
Cerebrovascular disease	1,260	72.2%	157	69.4%	262	76.0%	87	69.0%	145	69.7%
Chronic pulmonary disease	4,200	47.0%	494	43.3%	1,393	50.6%	244	38.5%	376	49.5%
Hypertension	8,940	48.8%	1,500	45.7%	1,444	57.1%	658	43.5%	1,021	48.4%
Diabetes	3,388	48.9%	564	47.2%	535	58.9%	216	40.3%	334	46.4%
Liver disease	531	37.5%	68	42.6%	75	32.0%	24	16.7%	15	53.3%
Renal disease	1,965	62.7%	303	64.7%	329	66.6%	87	62.1%	183	60.7%
Peptic ulcer	709	43.0%	110	40.9%	102	52.0%	32	21.9%	49	61.2%
Anaemia	1,373	46.9%	476	42.9%	161	59.0%	77	44.2%	58	60.3%
Neurodegenerative disorders	1,568	47.3%	199	53.3%	262	48.1%	123	40.7%	135	49.6%
Rheumatic disorders	574	50.3%	66	45.5%	124	49.2%	43	60.5%	41	51.2%

Among those diagnosed with cancer (ex NMSC) 22.6% had a previous diagnosis of cardiovascular disease in the past five-years, with this proportion greater among males than females (27.1% vs. 18.2%, *p*<0.001). The proportion of patients with pre-existing cardiovascular diseaseincreased with age (aged 0-54: 3.6%; aged 80+: 43.6%, p<0.001) and stage (stage I/II: 17.3%; stage IV: 24.2%, p<0.001), but did not vary significantly by deprivation (least deprived: 20.3%, most deprived: 21.3%, p=0.152). Among patients with the most common cancer types, 25.3% of colorectal cancer patients, 32.3% of lung cancer patients, 10.4% of female breast cancer patients and 22.6% of prostate cancer patients had pre-existing heart disease.

**Fig. 1:**
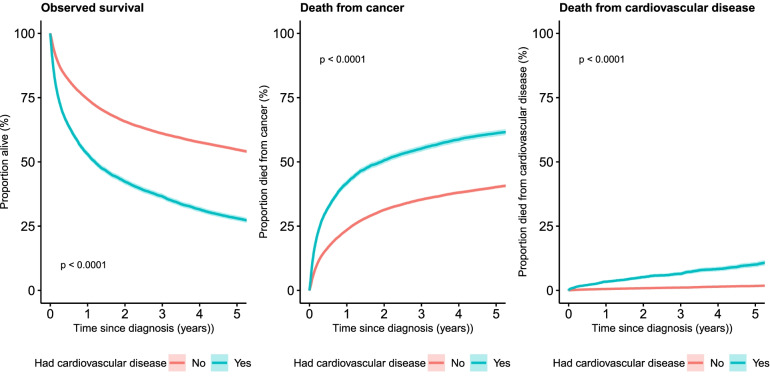
Cancer survival by presence of cardiovascular disease prior to diagnosis: All cancers (ex NMSC) diagnosed 2011-2014

Observed survival for patients with cancer (ex NMSC) and no pre-existing cardiovascular diseasewas 74.7% (95% CI: 74.2% - 75.2%) one year from diagnosis and 54.6% (95% CI: 54.0% - 55.2%) five years from diagnosis. This was significantly higher than the one-year observed survival of 53.6% (95% CI: 52.5% - 54.7%) and five-year survival of 28.2% (95% CI: 27.2% - 29.2%) for patients with pre-existing cardiovascular disease. The difference was reduced, but still significant, when cause of death was restricted to cancer only (five-year cancer specific survival: 58.8% vs 39.0% for patients without vs patients with pre-existing cardiovascular disease) as 10.0% of the cohort of patients with pre-existing cardiovascular diseasedied from cardiovascular disease. The group of patients without pre-existing cardiovascular diseasestill had a small proportion (1.7%) dying from a cardiovascular disease (Table 2, Fig. 1).

**Table 2:** Survival of cancer patients by cancer type and existence of a pre-existing cardiovascular disease: Patients diagnosed 2011-2014, followed up to the end of 2019

Survival time/type	Pre-existing cardiovascular diseases	All cancer (ex NMSC)	Colorectal cancer	Lung cancer	Female breast cancer	Prostate cancer
**One-year observed survival (95% CI)**	**No**	74.7% (74.2%, 75.2%)	79.9% (78.5%, 81.2%)	34.7% (33.0%, 36.4%)	95.7% (95.1%, 96.3%)	95.3% (94.5%, 95.9%)
	**Yes**	53.6% (52.5%, 54.7%)	68.1% (65.3%, 70.7%)	27.6% (25.4%, 29.9%)	83.9% (80.4%, 86.8%)	86.8% (84.5%, 88.8%)
**Five-year observed survival (95% CI)**	**No**	54.6% (54.0%, 55.2%)	56.1% (54.4%, 57.7%)	11.4% (10.3%, 12.5%)	80.5% (79.3%, 81.6%)	77.1% (75.6%, 78.5%)
	**Yes**	28.2% (27.2%, 29.2%)	37.7% (34.9%, 40.5%)	6.4% (5.3%, 7.8%)	48.7% (44.3%, 53.0%)	56.5% (53.3%, 59.6%)
**One-year cancer specific survival (95% CI)**	**No**	76.9% (76.3%, 77.4%)	82.0% (80.7%, 83.3%)	36.9% (35.2%, 38.6%)	96.5% (95.9%, 97.0%)	96.9% (96.3%, 97.5%)
	**Yes**	58.8% (57.7%, 60.0%)	74.6% (71.9%, 77.0%)	30.9% (28.5%, 33.3%)	89.2% (86.1%, 91.6%)	91.8% (89.9%, 93.4%)
**Five-year cancer specific survival (95% CI)**	**No**	59.6% (59.0%, 60.2%)	60.8% (59.2%, 62.4%)	13.8% (12.6%, 15.1%)	85.0% (83.9%, 86.0%)	83.8% (82.5%, 85.1%)
	**Yes**	39.0% (37.8%, 40.2%)	50.9% (47.8%, 54.0%)	9.3% (7.8%, 11.1%)	70.1% (65.6%, 74.2%)	73.1% (70.0%, 76.0%)
**Proportion who died from** cardiovascular diseases **after five-years (95% CI)**	**No**	1.7% (1.5%, 1.9%)	1.8% (1.4%, 2.5%)	3.4% (2.2%, 5.1%)	1.1% (0.8%, 1.5%)	1.9% (1.4%, 2.4%)
	**Yes**	10.0% (9.0%, 11.0%)	9.7% (7.7%, 12.2%)	9.8% (6.8%, 14.2%)	10.8% (8.0%, 14.4%)	8.0% (6.2%, 10.2%

*CI* Confidence interval

Note: Observed survival uses deaths from any cause as the vital event, while cancer-specific survival uses deaths from cancer only, with patients who dies from other causes censored at the time of death

**Fig. 2:**
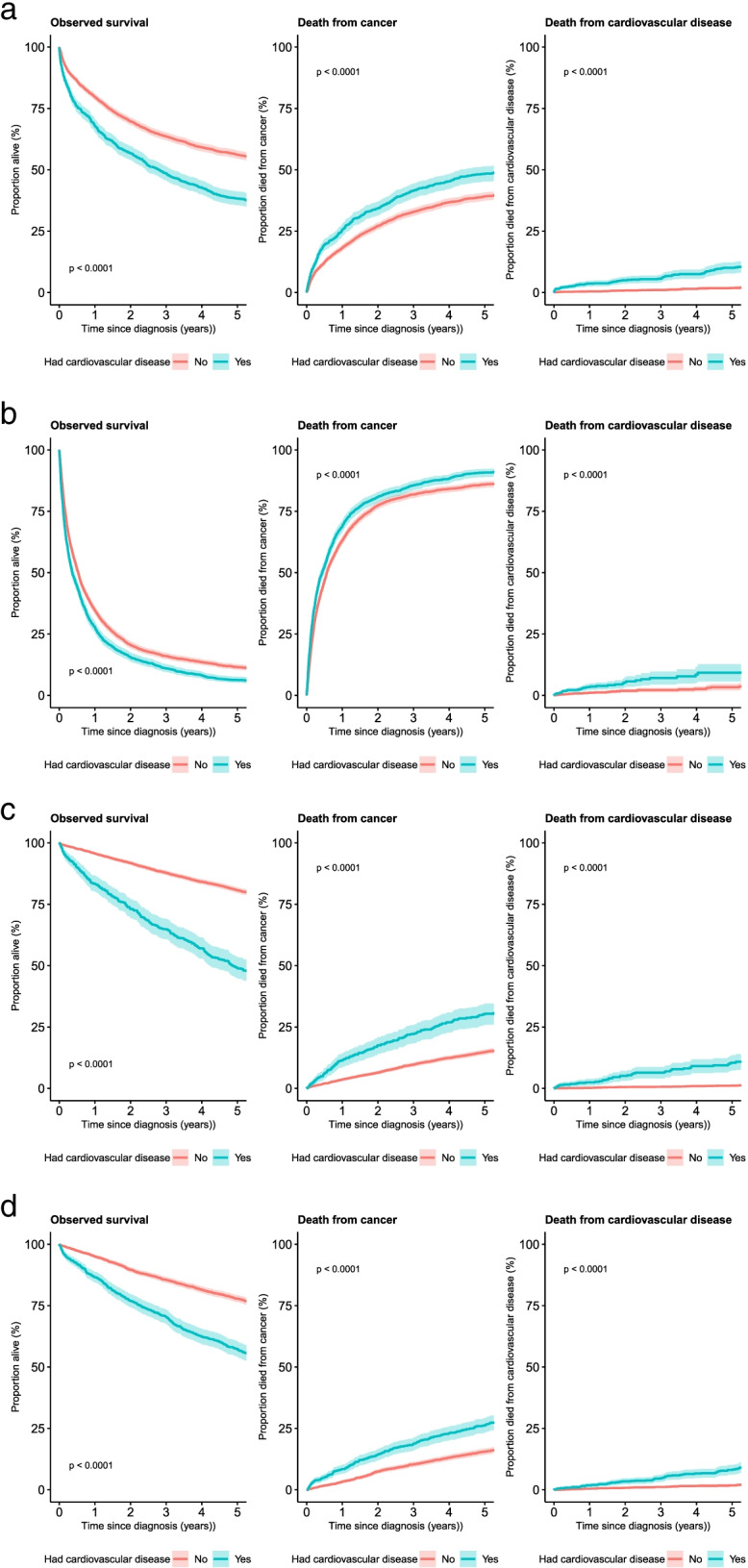
Cancer survival by presence of cardiovascular disease prior to diagnosis by cancer type. (**a**) Colorectal cancer diagnosed 2011-2014. (**b**) Lung cancer diagnosed 2011-2014. (**c**) Female breast cancer diagnosed 2011-2014. (**d**) Prostate cancer diagnosed 2011-2014

A similar pattern was present among patients with the four most common cancers. Among colorectal cancer patients there was a statistically significant 18.4% difference in five-year observed survival between those with and without pre-existing cardiovascular disease. Similarly for lung cancer patients the difference was 5.0%, for female breast cancer it was 31.8% and for prostate cancer it was 20.8%. Considering cancer-specific survival, the differences between patient cohorts reduced, but were still statistically significant (Table 2, Fig. 2).

After adjusting for age, stage, gender and comorbidities, the hazard ratio comparing all causes of survival of patients with and without cardiovascular disease was 1.36 (95% CI: 1.26 – 1.46). This reduced to 1.28 (95% CI: 1.18 - 1.40) when only cancer death was considered. Similarly, across the four common cancer types, patients with a pre-existing cardiovascular disease were more likely to die sooner than those without, regardless of whether all cause or cancer specific mortality is considered. All differences were statistically significant, except for cause-specific survival in colorectal cancer (Table 3).

**Table 3:** Adjusted cancer patient survival hazard ratio for patients with cardiovascular disease prior to diagnosis compared to those with no prior history of cardiovascular disease: Patients diagnosed 2011-2014, followed up to the end of 2019

Cancer type	Adjusted hazard ratio (95% CI)^a^ – cardiovascular disease prior to diagnosis vs. no prior history of cardiovascular diseases	
	Observed survival (i.e. death from any cause)	Cancer-specific survival (i.e. death from cancer only)
All cancers (ex NMSC)	1.36 (1.26, 1.46)	1.28 (1.18, 1.40)
Colorectal cancer	1.26 (1.03, 1.53)	1.15 (0.91, 1.45)
Lung cancer	1.35 (1.13, 1.60)	1.28 (1.07, 1.54)
Female breast cancer	1.42 (1.03, 1.94)	1.51 (1.01, 2.26)
Prostate cancer	1.52 (1.19, 1.94)	1.74 (1.28, 2.35)

*CI* Confidence interval

^a^Adjusted for gender, age group, stage at diagnosis, area-based deprivation and other comorbidity. An interaction term between heart disease and deprivation is included.

*Note* The full models are available in Supplementary table 1

The full results of the multivariate models are available in Supplementary table 1. They show considerable variation in survival by increasing age and stage at diagnosis along with small, but significant, variation in survival by deprivation and specific comorbidities other than cardiovascular disease. For example having a previous cancer results in poorer cancer-specific survival for all cancers (ex NMSC) and for colorectal, female breast and prostate cancer.

## Discussion

To improve cancer outcomes it is necessary to have a clear understanding of the role relevant factors, some of which may not be amenable to intervention, but which can affect understanding of international survival differences. We have documented in a large group of cancer patients that similar to other studies those jurisdictions with a higher burden of comorbidity have poorer survival compared to those with a lesser burden likely attributable to individual patient resilience to toxicity and/or eligibility to treatment. Identifying the role of comorbidity not only contributes to our understanding of differences in cancer outcomes but may also help in the development of interventions to improve outcomes, for example, early identification and treatment of comorbidities at the point of a cancer diagnosis. Indeed given the relationship between multi-morbidity and age, this work may raise awareness amongst clinicians and serve as evidence for facilitating conversations around treatment options and outcome expectations, and around the importance of secondary prevention.

The prevalence of pre-existing cardiovascular disease found in this study is similar to that reported by Strongman et al [28] for the UK at around 30% but notably much higher than that reported by Youn et al [26] (11.3%) for Korea. As with Youn et al, we demonstrate a clear penalty for both cancer specific and all-cause mortality associated with pre-existing cardiovascular disease. Given the different populations, prevalence of cardiovascular disease and range of variables controlled for in the analyses (Youn et al do not control for staging) care is warranted in comparing findings between these studies. That said, the adjusted hazard ratio (aHR) for 5 year all-cause mortality in Youn et al was 1.31 (95% CI1.19, 1.44) which is close to that found here at 1.28 (95% CI: 1.18 - 1.40) in our fully adjusted model. Given the higher prevalence of cardiovascular disease in NI , noted above, the penalty attached at a population level could well be expected to impact on outcomes compared to those achieved in other jurisdictions with a lower prevalence of pre-existing cardiovascular disease or indeed of comorbidity more generally. Other factors, of course, are also likely to contribute to differences in outcomes, including access to timely and optimal care as well as differences in patterns of screening. A lower recorded incidence of prostate cancer in Northern Ireland may in part, for example, be grounded in lower levels of PSA testing leading to a lower prevalence of non-life threatening cancers resulting in inflated statistics compared to some other jurisdictions.

With respect to the specific cancers data we were able to examine, the results show distinct patterns in the role of pre-existing cardiovascular disease for all cause and cancer specific mortality. A higher aHR with respect to lung, breast and prostate cancer is evident than for colorectal cancer in both cases, for example, while the cancer specific aHR for prostate cancer and breast are higher than that for all cause. The difference between cancer and all-cause specific aHRs may reflect a greater penalty related to access to optimal treatment in these cancers compared to colorectal. For example, pre-existing cardiovascular disease may be more impactful on survival in prostate than colorectal cancer, possibly related to differences in the overall frailty, competing risks of death or due to cardiotoxicity associated with endocrine therapies utilised. This may similarly be the case with respect to differences across cancers with respect aHR within cancer specific mortality.

The differences found with respect to other covariates are consistent with expectations supporting the face validity of these results. For example, as age increase so too do aHRs, as also seen in stage at diagnosis and deprivation. With respect to other morbidities, again results are consistent with expectations these generally increasing aHR for cancer specific and all-cause mortality. That there is not a clear pattern in interaction terms between pre-existing cardiovascular disease and death when other variables are controlled suggests an absence of inequalities with respect to the specific role of cardiovascular disease in mortality.

The results overall underscore the importance of controlling for comorbidities and pre-existing cardiovascular disease in particular when examining cancer survival. Whether pre-existing cardiovascular disease is causally related to survival is not possible to determine from our analysis. That (as suggested by some) systemic inflammation may play a primary role in both heart disease and malignancy is an intriguing possibility. Understanding the contribution of comorbidity is clearly important to the interpretation of survival statistics and the development of appropriate policy responses.

The data presented will inform 'cardiooncology' services that have been established in tertiary centres globally in recent years [34]. Such dedicated teams, composed of cardiologists, oncologists and specialist nurses, have been instigated to both provide specialist clinical care at the interface of these two specialties, and to facilitate interdisciplinary education. The published early UK experience of this type of service showcased the caseload and potential clinical benefit as well as making recommendations for how cardiooncology clinics can be developed [35]. Recent data indicate that variation in access to focussed cardiooncology clinics contributes to the recognised disparity in cardiovascular outcomes in patients with cancer [36]. Such reports can be found in novel specialist journals established by reputable publishers in order to facilitate the dissemination of research in this maturing field [37, 38]. Following a recent global cardiooncology summit, priority research areas for enhancing clinical cardiac outcomes were agreed upon, spanning risk prediction, preventative agents and personalised approaches [36] (Lenihan 2019). Research-driven advancement of the cardiooncology field would be highly valuable, and there are plans for the data presented to be incorporated in the future refinement of the cardiooncology service in Northern Ireland.

There are a number of limitations to our study. First, we are not able to control for several variables that have been shown to impact on survival in other studies including smoking status and BMI. While this is conceded, the comprehensive list of comorbid conditions controlled for including COPD, diabetes, and renal disease does offer a mechanism by which elevated risks related to these may be incorporated into our analyses. Second, we do not examine treatments received for cancer care in this study due to a lack of data availability. These would have allowed us to examine the role of pre-existing cardiovascular disease on access to care as intermediate outcomes, and permitted controlling for them when seeking to understand the role of pre-existing cardiovascular disease (and other comorbid conditions). These data were not available to us, however. Third, an examination of specific heart conditions and survival may have allowed us to shed additional light on relationships, whether for example heart failure played a different role to arrhythmias. Although it does warrant examination in larger populations, within the context of the relatively small population herein, it was not feasible to undertake this more granular analysis. Fourth, we were able to identify cardiovascular disease up to five years prior to cancer diagnoses based on treatment patterns. Should a person have not received treatment in this window we would have assumed they did not have a heart condition. Fifth, treatment patterns were based on observed use of hospital services. It is conceivable that individuals whose cardiovascular disease is managed exclusively in the community, or whose condition is undiagnosed, remain unobserved and misclassified as unaffected by cardiovascular disease. Sixth, while we have examined all cause and cause specific mortality we acknowledge the possibility of misclassification of specific causes of death. While we have assumed any misclassification is random and would not therefore affect results, this may not be the case.

## Conclusions

We show a high prevalence of pre-existing heart disease among cancer patients in NI. We demonstrate that pre-existing heart disease is associated with a significant survival penalty across all cancers combined when controlling for a range of covariates as well as with respect to all-cause mortality. Our findings have implications for the management of cancer patients with heart disease, for public health measures intended to reduce multi-morbidity in general and improve heart health in particular. More specifically, they also have implications for service planning as the interaction between cardiology and oncology grows.

## Supplementary Information

**Additional file 1** .

## References

[1] Sant M, Capocaccia M, Coleman MP, Berrino F, Gatta G, Micheli A (2001). Cancer survival increases in Europe, but international differences remain wide. European Journal of Cancer.

[2] Qiu H, Cao S, Xu R (2021). Cancer incidence, mortality, and burden in China: a time-trend analysis and comparison with the United States and United Kingdom based on the global epidemiological data released in 2020. Cancer Commun (Lond)..

[3] Coleman MP, Forman D, Bryant H (2011). Cancer survival in Australia, Canada, Denmark, Norway, Sweden, and the UK, 1995-2007 (the International Cancer Benchmarking Partnership): an analysis of population-based cancer registry data. Lancet.

[4] Araghi M, Arnold M, Rutherford MJ, Guren MG, Cabasag CJ, Bardot A (2021). Colon and rectal cancer survival in seven high-income countries 2010–2014: variation by age and stage at diagnosis (the ICBP SURVMARK-2 project). Gut.

[5] Wong, M.C.S., Lao, X.Q., Ho, KF. et al*.* Incidence and mortality of lung cancer: global trends and association with socioeconomic status. Sci Rep 7, 14300 (2017). https://doi.org/10.1038/s41598-017-14513-7 10.1038/s41598-017-14513-7 PMC5662733 29085026

[6] DeSantis CE, Bray F, Ferlay J, Lortet-Tieulent J, Anderson BO, Jemal A. International Variation in Female Breast Cancer Incidence and Mortality Rates. Cancer Epidemiol Biomarkers Prev. 2015 24(10):1495-1506. doi: 10.1158/1055-9965.EPI-15-0535. Epub 2015 Sep 10. PMID: 26359465. 10.1158/1055-9965.EPI-15-0535 26359465

[7] Taitt HE (2018). Global Trends and Prostate Cancer: A Review of Incidence, Detection, and Mortality as Influenced by Race, Ethnicity, and Geographic Location. Am J Mens Health.

[8] Walters S, Maringe C, Coleman MP (2013). Lung cancer survival and stage at diagnosis in Australia, Canada, Denmark, Norway, Sweden and the UK: a population-based study, 2004-2007. Thorax.

[9] Singh H, Hirani K, Kadiyala H (2010). Characteristics and predictors of missed opportunities in lung cancer diagnosis: an electronic health record-based study. J Clin Oncol.

[10] Møller H, Richards S, Hanchett N (2011). Completeness of case ascertainment and survival time error in English cancer registries: impact on 1-year survival estimates. Br J Cancer.

[11] Rutherford MJ, Møller H, Lambert PC (2013). A comprehensive assessment of the impact of errors in the cancer registration process on 1- and 5-year relative survival estimates. Br J Cancer.

[12] Woods LM, Coleman MP, Lawrence G (2011). Evidence against the proposition that “UK cancer survival statistics are misleading”: simulation study with National Cancer Registry data. BMJ.

[13] Northern Ireland Cancer Registry 2021. https://www.qub.ac.uk/research-centres/nicr/CancerInformation/official-statistics/. Accessed 1 Aug 2022.

[14] Lüchtenborg, M., Morris, E. J. A., Tataru, D., Coupland, V. H., Smith, A., Milne, R. L., Te Marvelde, et al . Investigation of the international comparability of population-based routine hospital data set derived comorbidity scores for patients with lung cancer. Thorax, 2017. 73(4), 339-349. https://doi.org/10.1136/thoraxjnl-2017-210362 10.1136/thoraxjnl-2017-210362 PMC5870453 29079609

[15] Søgaard M, Thomsen RW, Bossen KS, Sørensen HT, Nørgaard M (2013). The impact of comorbidity on cancer survival: a review. Clin Epidemiol..

[16] Imperatori A, Harrison RN, Leitch DN, Rovera F, Lepore G, Dionigi G, Sutton P, Dominioni L (2006). Lung cancer in Teesside (UK) and Varese (Italy): a comparison of management and survival. Thorax.

[17] Cronin-Fenton DP, Nørgaard M, Jacobsen J (2007). Comorbidity and survival of Danish breast cancer patients from 1995 to 2005. Br J Cancer..

[18] Iversen LH, Nørgaard M, Jacobsen J, Laurberg S, Sørensen HT (2009). The impact of comorbidity on survival of Danish colorectal cancer patients from 1995 to 2006 – a population-based cohort study. Dis Colon Rectum..

[19] Asmis TR, Ding K, Seymour L (2008). National Cancer Institute of Canada Clinical Trials Group. Age and comorbidity as independent prognostic factors in the treatment of non small-cell lung cancer:a review of National Cancer Institute of Canada Clinical Trials Group trials. J Clin Oncol..

[20] Houterman S, Janssen-Heijnen ML, Verheij CD (2004). Comorbidity has negligible impact on treatment and complications but influences survival in breast cancer patients. Br J Cancer..

[21] Janssen-Heijnen ML, Houterman S, Lemmens VE, Louwman MW, Maas HA, Coebergh JW (2005). Prognostic impact of increasing age and co-morbidity in cancer patients: a population-based approach. Crit Rev Oncol Hematol..

[22] Carney P, Gavin A, O'Neill C (2013). The role of private care in the interval between diagnosis and treatment of breast cancer in Northern Ireland: an analysis of Registry data. BMJ Open..

[23] Bradley CJ, Given CW, Roberts C (2002). Race, socioeconomic status, and breast cancer treatment and survival. J Natl Cancer Inst.

[24] Wu X-C, Lund MJ, Kimmick GG (2012). Influence of race, insurance, socioeconomic status, and hospital type on receipt of guideline concordant adjuvant systemic therapy for locoregional breast cancers. J Clin Oncol.

[25] Fang P, He W, Gomez D (2018). Racial disparities in guideline-concordant cancer care and mortality in the United States. Adv Radiat Oncol.

[26] Youn JC, Chung WB, Ezekowitz JA, Hong JH, Nam H, Kyoung DS, Kim IC, Lyon AR, Kang SM, Jung HO, Chang K, Oh YS, Youn HJ, Baek SH, Kim HC (2020). Cardiovascular disease burden in adult patients with cancer: An 11-year nationwide population-based cohort study. Int J Cardiol..

[27] Bertero E, Canepa M, Maack C, Ameri P (2018). Linking Heart Failure to Cancer: Background Evidence and Research Perspectives. Circulation..

[28] Strongman H, Gadd S, Matthews A, Mansfield KE, Stanway S, Lyon AR, Dos-Santos-Silva I, Smeeth L, Bhaskaran K (2019). Medium and long-term risks of specific cardiovascular diseases in survivors of 20 adult cancers: a population-based cohort study using multiple linked UK electronic health records databases. Lancet.

[29] Charlson ME, Pompei P, Ales KL (1987). A new method of classifying prognostic comorbidity in longitudinal studies: development and validation. J Chronic Dis.

[30] Elixhauser A, Steiner C, Harris DR (1998). Comorbidity measures for use with administrative data. Med Care.

[31] Northern Ireland Statistics and Research Agency. Northern Ireland Multiple Deprivation Measure 2017. Available at https://www.nisra.gov.uk/statistics/deprivation/northern-ireland-multiple-deprivation-measure-2017-nimdm2017#toc-0. Accessed Apr 2021.

[32] R Core Team. R. A language and environment for statistical computing. R Foundation for Statistical Computing. Vienna. Available at https://www.R-project.org/. Accessed Apr 2021.

[33] Kassambara A, Kosinski M, Biecek P. survminer: Drawing survival curves using 'ggplot2'. R package version 0.4.9. Available at https://CRAN.R-project.org/package=survminer. Accessed Apr 2021.

[34] Bhatt DL (2019). Birth and Maturation of CardioOncology. JACC CardioOncol..

[35] Pareek N, Cevallos J, Moliner P, Shah M, Tan LL, Chambers V, Baksi AJ, Khattar RS, Sharma R, Rosen SD, Lyon AR (2018). Activity and outcomes of a cardiooncology service in the United Kingdom-a five-year experience. Eur J Heart Fail..

[36] Lenihan DJ, Fradley MG, Dent S, Brezden-Masley C, Carver J, Filho RK, Neilan TG, Blaes A, Melloni C, Herrmann J, Armenian S, Thavendiranathan P, Armstrong GT, Ky B, Hajjar L (2019). Proceedings From the Global CardioOncology Summit: The Top 10 Priorities to Actualize for CardioOncology. JACC CardioOncol..

[37] Lipshultz SE, Minotti G, Carver J, Franco VI (2015). An Invitation from the Editors of CardioOncology. Cardiooncology..

[38] Ky B (2019). JACC: CardioOncology: Poised to Serve a Maturing, Collaborative Field. JACC CardioOncol.

