# Supplementary material for: Survival of cancer patients with pre-existing heart disease
Source: BMC Cancer. 2022 Aug 3;22:847. doi: 10.1186/s12885-022-09944-z (PMC9351236; doi:10.1186/s12885-022-09944-z)
Supplement: Supplementary file 1 — Additional file 1 . [file 12885_2022_9944_MOESM1_ESM.docx]

## Supplementary material: Comorbidity classification

## Heart disease classification

Heart disease was identified using ICD10 codes from the hospital episodes dataset as follows:

- Congestive heart failure: I09.9, I11.0, I13.0, I13.2, I25.5, I42.0, I42.5, I42.6, I42.7, I42.8, I42.9, I43.x, I50.x, P29.0
- Peripheral vascular disease: I67.x, I70.x, I71.x, I73.1, I73.8, I73.9, I77.1, I79.0, I79.2, K55.1, K55.8, K55.9, Z95.8, Z95.9
- Atrial fibrillation: I48.x
- Cardiac arrhythmias (ex Atrial fibrillation): I44.1, I44.2, I44.3, I45.6, I45.9, I47.x, I49.x, R00.0, R00.1, R00.8, T82.1, Z45.0, Z95.0
- Valvular disease: A52.0, I05.x, I09.1, I09.8, I34.x, I35.x, I36.x, I37.x, I38.x, I39.x, Q23.0, Q23.1, Q23.2, Q23.3, Z95.2, Z95.3, Z95.4
- Pulmonary circulation disorder: I26.x, I27.x, I28.0, I28.8, I28.9
- Myocardial infarction: I21.x, I22.x, I25.2
- Other ischaemic heart diseases (excluding myocardial infarction): I20.x, I25.0, I25.1, I25.3, I25.4, I25.5, I25.6, I25.8, I25.9
- Venous or arterial embolism and thrombosis: I74.x, I82.x
- Myocarditis & Pericarditis: I30.x, I31.x, I32.x, I40.x, I41.x
- Cardiac arrest: I46.x

Any heart disease is defined by having one or more of the above.

## Classification of other comorbidities

All other conditions were identified using ICD10 codes from the hospital episodes dataset as follows:

- Cerebrovascular disease: G45.x, G46.x, H34.0, I60.x, I61.x, I62.x, I63.x, I64.x, I65.x, I66.x, I67.x, I68.x, I69.x
- Chronic pulmonary disease: I27.8, I27.9, J40.x, J41.x, J42.x, J43.x, J44.x, J45.x, J46.x, J47.x, J60.x, J61.x, J62.x, J63.x, J64.x, J65.x, J66.x, J67.x, J68.4, J70.1, J70.3
- Hypertension: I10.x, I11.x, I12.x, I13.x, I15.x
- Diabetes: E10.x, E11.x, E12.x, E13.x, E14.x
- Liver disease: B18.x, I85.0, I85.9, I86.4, I98.2, K70.0, K70.1, K70.2, K70.3, K70.4, K70.9, K71.1, K72.1, K71.3, K71.4, K71.5, K71.7, K72.9, K73.x, K74.x, K76.0, K76.2, K76.3, K76.4, K76.5, K76.6, K76.7, K76.8, K76.9, Z94.4
- Renal disease: I12.0, I13.1, N03.2, N03.3, N03.4, N03.5, N03.6, N03.7, N05.2, N05.3, N05.4, N05.5, N05.6, N05.7, N18.x, N19.x, N25.0, Z49.0, Z49.1, Z49.2, Z94.0, Z99.2
- Peptic ulcer: K25.x, K26.x, K27.x, K28.x
- Anaemia: D50.x, D51.x, D52.x, D53.x, D62.x
- Neurodegenerative disorders: F00.x, F01.x, F02.x, F03.x, F05.1, G10.x, G11.0, G11.1, G11.2, G11.3, G11.8, G11.9, G12.x, G13.x, G20.x, G22.x, G25.4, G25.5, G30.x, G31.1, G31.2, G31.8, G31.9, G32.x, G35.x, G37.x, G40.x, G41.x, G93.1, G93.4, R47.0, R56.x
- Rheumatoid disorders: L94.0, L94.1, L94.3, M05.x, M06.x, M08.x, M12.0, M12.3, M30.x, M31.0, M31.1, M31.2, M31.3, M32.x, M33.x, M34.x, "M35.x, M45.x, M46.1, M46.8, M46.9

## Supplementary table: Multivariable Cox regression modelling patient survival after cancer diagnosis by cancer type: All cancers (ex NMSC) diagnosed 2011-2014

*(a) Observed survival (i.e. death from any cause)*

| **Characteristic** | **Hazard ratio (95% CI)** | | | | |
| --- | --- | --- | --- | --- | --- |
|  | **All cancers (ex NMSC)** | **Colorectal**  **cancer** | **Lung**  **cancer** | **Female breast**  **cancer** | **Prostate**  **cancer** |
| **Heart condition** |  |  |  |  |  |
| No heart condition | 1.00 | 1.00 | 1.00 | 1.00 | 1.00 |
| Had heart condition | 1.36  (1.26, 1.46) | 1.26  (1.03, 1.53) | 1.35  (1.13, 1.60) | 1.42  (1.03, 1.94) | 1.52  (1.19, 1.94) |
|  |  |  |  |  |  |
| **Gender** |  |  |  |  |  |
| Male | 1.00 | 1.00 | 1.00 |  |  |
| Female | 1.06  (1.03, 1.09) | 0.95  (0.88, 1.04) | 0.89  (0.83, 0.94) |  |  |
|  |  |  |  |  |  |
| **Age group** |  |  |  |  |  |
| Age 0-54 | 1.00 | 1.00 | 1.00 | 1.00 | 1.00 |
| Age 55-69 | 1.70  (1.61, 1.80) | 1.28  (1.08, 1.50) | 1.40  (1.22, 1.60) | 1.20  (1.01, 1.43) | 1.09  (0.74, 1.61) |
| Age 70-79 | 2.52  (2.39, 2.66) | 2.06  (1.75, 2.41) | 1.75  (1.53, 2.00) | 2.42  (2.05, 2.86) | 2.17  (1.48, 3.19) |
| Age 80+ | 3.99  (3.78, 4.23) | 4.17  (3.53, 4.92) | 2.40  (2.08, 2.76) | 4.53  (3.82, 5.38) | 4.98  (3.38, 7.36) |
|  |  |  |  |  |  |
| **Deprivation** |  |  |  |  |  |
| Least deprived | 1.00 | 1.00 | 1.00 | 1.00 | 1.00 |
| Quintile 2 | 1.10  (1.04, 1.16) | 1.10  (0.94, 1.29) | 1.10  (0.96, 1.26) | 1.01  (0.84, 1.22) | 1.27  (1.04, 1.55) |
| Quintile 3 | 1.10  (1.04, 1.17) | 1.14  (0.98, 1.33) | 1.10  (0.96, 1.25) | 0.93  (0.77, 1.13) | 1.11  (0.91, 1.35) |
| Quintile 4 | 1.19  (1.13, 1.26) | 1.24  (1.06, 1.45) | 1.16  (1.02, 1.32) | 1.13  (0.94, 1.35) | 1.13  (0.92, 1.38) |
| Most deprived | 1.46  (1.38, 1.54) | 1.26  (1.08, 1.47) | 1.29  (1.14, 1.45) | 1.29  (1.07, 1.55) | 1.28  (1.03, 1.59) |
|  |  |  |  |  |  |
| **Stage** |  |  |  |  |  |
| Stage I/II | 1.00 | 1.00 | 1.00 | 1.00 | 1.00 |
| Stage III | 2.74  (2.62, 2.88) | 2.18  ( 1.94, 2.45) | 2.31  (2.10, 2.55) | 3.32  (2.88, 3.83) | 1.21  (1.00, 1.46) |
| Stage IV | 8.19  (7.86, 8.54) | 12.95  (11.55, 14.52) | 4.90  (4.48, 5.36) | 12.06  (10.33, 14.08) | 6.42  (5.62, 7.34) |
| Unknown | 4.28  (4.11, 4.46) | 5.91  (5.18, 6.73) | 3.22  (2.88, 3.59) | 3.41  (2.89, 4.02) | 3.64  (3.10, 4.28) |
|  |  |  |  |  |  |
| **Comorbidity** |  |  |  |  |  |
| No previous cancer | 1.00 | 1.00 | 1.00 | 1.00 | 1.00 |
| Previous cancer | 1.21  (1.14, 1.29) | 1.28  (1.10, 1.49) | 0.87  (0.76, 0.98) | 1.46  (1.18, 1.81) | 1.82  (1.42, 2.33) |
| No COPD | 1.00 | 1.00 | 1.00 | 1.00 | 1.00 |
| COPD | 1.42  (1.36, 1.47) | 1.04  (0.92, 1.17) | 1.05  (0.98, 1.12) | 1.22  ( 0.98, 1.50) | 1.46  (1.25, 1.72) |
| No cerebrovascular disease | 1.00 | 1.00 | 1.00 | 1.00 | 1.00 |
| Cerebrovascular disease | 1.16  (1.09, 1.24) | 1.13  (0.92, 1.37) | 1.21  (1.06, 1.39) | 1.33  (1.00, 1.77) | 1.20  (0.95, 1.50) |
| No hypertension | 1.00 | 1.00 | 1.00 | 1.00 | 1.00 |
| Hypertension | 1.01  (0.97, 1.04) | 0.95  (0.87, 1.04) | 0.98  (0.91, 1.06) | 1.03  (0.88, 1.20) | 1.03  (0.91, 1.17) |
| No diabetes | 1.00 | 1.00 | 1.00 | 1.00 | 1.00 |
| Diabetes | 1.08  (1.03, 1.13) | 1.06  (0.94, 1.20) | 0.98  (0.89, 1.08) | 1.04  (0.84, 1.28) | 1.18  (1.00, 1.40) |
| No liver disease | 1.00 | 1.00 | 1.00 | 1.00 | 1.00 |
| Liver disease | 1.66  (1.51, 1.82) | 1.02  (0.77, 1.34) | 1.34  (1.06, 1.70) | 2.61  (1.51, 4.50) | 1.25  (0.64, 2.44) |
| No renal disease | 1.00 | 1.00 | 1.00 | 1.00 | 1.00 |
| Renal disease | 1.36  (1.29, 1.44) | 1.33  (1.16, 1.54) | 1.17  (1.04, 1.32) | 2.07  (1.58, 2.72) | 1.69  (1.39, 2.04) |
| No peptic ulcer | 1.00 | 1.00 | 1.00 | 1.00 | 1.00 |
| Peptic ulcer | 1.33  (1.22, 1.45) | 1.06  (0.84, 1.34) | 1.33  (1.09, 1.63) | 1.38  (0.79, 2.41) | 1.07  (0.71, 1.60) |
| No anaemia | 1.00 | 1.00 | 1.00 | 1.00 | 1.00 |
| Anaemia | 1.08  (1.01, 1.15) | 1.03  (0.91, 1.17) | 1.31  (1.11, 1.55) | 1.25  (0.92, 1.71) | 1.03  (0.72, 1.45) |
| No neurodegenerative disorder | 1.00 | 1.00 | 1.00 | 1.00 | 1.00 |
| Neurodegenerative disorder (inc.dementia) | 1.50  (1.42, 1.60) | 1.37  (1.15, 1.62) | 1.23  (1.08, 1.41) | 1.92  (1.51, 2.45) | 2.35  (1.88, 2.93) |
| No rheumatic disease | 1.00 | 1.00 | 1.00 | 1.00 | 1.00 |
| Rheumatic disease | 1.16  (1.06, 1.28) | 0.63  (0.45, 0.89) | 0.98  (0.81, 1.18) | 1.21  (0.80, 1.83) | 1.36  (0.92, 2.01) |
|  |  |  |  |  |  |
| **Heart disease & deprivation** |  |  |  |  |  |
| Heart condition, Quintile 2 | 0.89  (0.80, 0.98) | 1.05  (0.81, 1.38) | 0.84  (0.67, 1.06) | 0.92  (0.61, 1.39) | 0.61  (0.44, 0.86) |
| Heart condition, Quintile 3 | 1.00  (0.91, 1.10) | 1.02  (0.78, 1.33) | 0.89  (0.72, 1.11) | 1.14  (0.75, 1.73) | 0.77  (0.55, 1.08) |
| Heart condition, Quintile 4 | 0.90  (0.82, 1.00) | 0.92  (0.70, 1.21) | 0.83  (0.67, 1.03) | 0.91  (0.61, 1.36) | 1.02  (0.73, 1.43) |
| Heart condition,  Most deprived | 0.84  (0.76, 0.93) | 1.02  (0.76, 1.36) | 0.75  (0.60, 0.92) | 0.99  (0.64, 1.53) | 0.87  (0.60, 1.25) |

*(b) Cancer-specific survival (i.e. death from cancer only)*

| **Characteristic** | **Hazard ratio (95% CI)** | | | | |
| --- | --- | --- | --- | --- | --- |
|  | **All cancers (ex NMSC)** | **Colorectal**  **cancer** | **Lung**  **cancer** | **Female breast**  **cancer** | **Prostate**  **cancer** |
| **Heart condition** |  |  |  |  |  |
| No heart condition | 1.00 | 1.00 | 1.00 | 1.00 | 1.00 |
| Had heart condition | 1.28  (1.18, 1.40) | 1.15  (0.91, 1.45) | 1.28  (1.07, 1.54) | 1.51  (1.01, 2.26) | 1.74  (1.28, 2.35) |
|  |  |  |  |  |  |
| **Gender** |  |  |  |  |  |
| Male | 1.00 | 1.00 | 1.00 |  |  |
| Female | 1.11  (1.07, 1.15) | 1.02  (0.93, 1.11) | 0.88  (0.82, 0.94) |  |  |
|  |  |  |  |  |  |
| **Age group** |  |  |  |  |  |
| Age 0-54 | 1.00 | 1.00 | 1.00 | 1.00 | 1.00 |
| Age 55-69 | 1.59  (1.50, 1.68) | 1.21  (1.02, 1.44) | 1.37  (1.19, 1.57) | 1.06  (0.87, 1.28) | 0.89  (0.58, 1.38) |
| Age 70-79 | 2.18  (2.06, 2.31) | 1.73  (1.47, 2.05) | 1.68  (1.46, 1.93) | 1.80  (1.50, 2.18) | 1.39  (0.91, 2.13) |
| Age 80+ | 3.13  (2.94, 3.32) | 3.49  (2.93, 4.16) | 2.30  (1.99, 2.67) | 2.60  (2.13, 3.19) | 3.14  (2.03, 4.86) |
|  |  |  |  |  |  |
| **Deprivation** |  |  |  |  |  |
| Least deprived | 1.00 | 1.00 | 1.00 | 1.00 | 1.00 |
| Quintile 2 | 1.09  (1.03, 1.16) | 1.13  (0.96, 1.34) | 1.10  (0.96, 1.27) | 0.96  (0.77, 1.19) | 1.14  (0.89, 1.47) |
| Quintile 3 | 1.10  (1.04, 1.17) | 1.15  (0.97, 1.36) | 1.11  (0.97, 1.28) | 0.97  (0.77, 1.21) | 0.95  (0.74, 1.22) |
| Quintile 4 | 1.21  (1.14, 1.29) | 1.22  (1.03, 1.45) | 1.20  (1.05, 1.37) | 1.14  (0.92, 1.41) | 1.19  (0.93, 1.53) |
| Most deprived | 1.46  (1.37, 1.54) | 1.23  (1.04, 1.45) | 1.33  (1.17, 1.51) | 1.29  (1.03, 1.61) | 1.28  (0.98, 1.67) |
|  |  |  |  |  |  |
| **Stage** |  |  |  |  |  |
| Stage I/II | 1.00 | 1.00 | 1.00 | 1.00 | 1.00 |
| Stage III | 3.55  (3.36, 3.74) | 3.06  (2.65, 3.55) | 2.66  (2.39, 2.96) | 4.36  (3.68, 5.16) | 1.58  (1.22, 2.07) |
| Stage IV | 11.40  (10.86, 11.96) | 21.47  (18.66, 24.69) | 5.82  (5.28, 6.42) | 19.17  (16.11, 22.80) | 13.41  (11.18, 16.10) |
| Unknown | 5.35  (5.09, 5.62) | 8.93  (7.61, 10.48) | 3.49  (3.09, 3.94) | 3.55  (2.84, 4.45) | 4.98  (3.94, 6.30) |
|  |  |  |  |  |  |
| **Comorbidity** |  |  |  |  |  |
| No previous cancer | 1.00 | 1.00 | 1.00 | 1.00 | 1.00 |
| Previous cancer | 1.28  (1.20, 1.36) | 1.25  (1.05, 1.49) | 0.88  (0.77, 1.00) | 1.94  (1.53, 2.46) | 2.66  (2.02, 3.51) |
| No COPD | 1.00 | 1.00 | 1.00 | 1.00 | 1.00 |
| COPD | 1.37  (1.32, 1.44) | 0.93  (0.81, 1.08) | 1.03  (0.96, 1.11) | 1.08  (0.81, 1.43) | 1.17  (0.93, 1.49) |
| No cerebrovascular disease | 1.00 | 1.00 | 1.00 | 1.00 | 1.00 |
| Cerebrovascular disease | 1.12  (1.04, 1.21) | 1.22  (0.97, 1.53) | 1.21  (1.05, 1.39) | 1.35  (0.91, 2.01) | 0.90  (0.63, 1.29) |
| No hypertension | 1.00 | 1.00 | 1.00 | 1.00 | 1.00 |
| Hypertension | 1.01  (0.97, 1.05) | 0.97  (0.88, 1.08) | 1.00  (0.93, 1.08) | 0.93  (0.76, 1.13) | 1.07  (0.91, 1.26) |
| No diabetes | 1.00 | 1.00 | 1.00 | 1.00 | 1.00 |
| Diabetes | 1.07  (1.02, 1.13) | 1.08  (0.94, 1.24) | 0.99  (0.89, 1.10) | 0.99  (0.75, 1.31) | 1.10  (0.88, 1.38) |
| No liver disease | 1.00 | 1.00 | 1.00 | 1.00 | 1.00 |
| Liver disease | 1.62  (1.46, 1.79) | 0.92  (0.67, 1.26) | 1.32  (1.03, 1.69) | 2.26  (1.11, 4.61) | 1.66  (0.73, 3.76) |
| No renal disease | 1.00 | 1.00 | 1.00 | 1.00 | 1.00 |
| Renal disease | 1.29  (1.21, 1.37) | 1.19  (1.00, 1.42) | 1.13  (0.99, 1.29) | 2.18  (1.52, 3.15) | 1.59  (1.22, 2.07) |
| No peptic ulcer | 1.00 | 1.00 | 1.00 | 1.00 | 1.00 |
| Peptic ulcer | 1.35  (1.22, 1.48) | 0.92  (0.69, 1.22) | 1.32  (1.06, 1.63) | 0.96  (0.40, 2.34) | 0.57  (0.27, 1.22) |
| No anaemia | 1.00 | 1.00 | 1.00 | 1.00 | 1.00 |
| Anaemia | 1.05  (0.97, 1.13) | 1.04  (0.90, 1.20) | 1.28  (1.07, 1.53) | 1.51  (1.03, 2.21) | 0.69  (0.38, 1.24) |
| No neurodegenerative disorder | 1.00 | 1.00 | 1.00 | 1.00 | 1.00 |
| Neurodegenerative disorder (inc.dementia) | 1.35  (1.26, 1.44) | 1.27  (1.04, 1.55) | 1.19  (1.03, 1.37) | 1.34  (0.94, 1.92) | 1.84  (1.31, 2.60) |
| No rheumatic disease | 1.00 | 1.00 | 1.00 | 1.00 | 1.00 |
| Rheumatic disease | 1.17  (1.05, 1.30) | 0.61  (0.41, 0.90) | 1.00  (0.82, 1.21) | 1.26  (0.74, 2.16) | 1.35  (0.79, 2.31) |
|  |  |  |  |  |  |
| **Heart disease & deprivation** |  |  |  |  |  |
| Heart condition, Quintile 2 | 0.90  (0.81, 1.01) | 0.96  (0.71, 1.32) | 0.88  (0.69, 1.12) | 0.80  (0.46, 1.39) | 0.49  (0.31, 0.76) |
| Heart condition, Quintile 3 | 1.01  (0.90, 1.13) | 0.91  (0.67, 1.24) | 0.94  (0.75, 1.18) | 0.90  (0.52, 1.56) | 0.67  (0.43, 1.04) |
| Heart condition, Quintile 4 | 0.87  (0.77, 0.97) | 0.89  (0.65, 1.22) | 0.82  (0.65, 1.03) | 0.69  (0.40, 1.19) | 0.74  (0.48, 1.14) |
| Heart condition,  Most deprived | 0.86  (0.77, 0.97) | 1.00  (0.71, 1.40) | 0.76  (0.61, 0.96) | 0.94  (0.54, 1.63) | 0.53  (0.32, 0.86) |
